# Tractography-Based Analysis of Morphological and Anatomical Characteristics of the Uncinate Fasciculus in Human Brains

**DOI:** 10.3390/brainsci10100709

**Published:** 2020-10-06

**Authors:** Sara Kierońska, Paweł Sokal, Marta Dura, Magdalena Jabłońska, Marcin Rudaś, Renata Jabłońska

**Affiliations:** 1 Department of Neurosurgery and Neurology, Jan Biziel University Hospital No 2, Collegium Medicum, Nicolaus Copernicus University, 85-168 Bydgoszcz, Poland; sara.kieronska@biziel.pl (S.K.); marcin.rudas@biziel.pl (M.R.); renata.jablonska@cm.umk.pl (R.J.); 2 Faculty of Health Science, The Ludwik Rydygier Collegium Medicum, Nicolaus Copernicus University, 85-067 Bydgoszcz, Poland; 3 Department of Radiology, Jan Biziel University Hospital No. 2, Collegium Medicum Nicolaus Copernicus University, 85-168 Bydgoszcz, Poland; marta.dura@cm.umk.pl; 4 Students’ Scientific Circle, Department of Neurosurgery and Neurology, Jan Biziel University Hospital No 2, Collegium Medicum, Nicolaus Copernicus University, 85-168 Bydgoszcz, Poland; magdalena.jablonska14@gmail.com; 5 Department of Neurological and Neurosurgical Nursing, Faculty of Health Science, The Ludwik Rydygier Collegium Medicum, Nicolaus Copernicus University, 85-067 Bydgoszcz, Poland

**Keywords:** uncinate fasciculus, tractography, fractional anisotropy, diffusion tensor imaging

## Abstract

(1) Background: The uncinate fasciculus (UF) is a white matter bundle connecting the prefrontal cortex and temporal lobe. The functional role of the uncinate fasciculus is still uncertain. The role of the UF is attributed to the emotional empathy network. The present study aimed to more accurately the describe anatomical variability of the UF by focusing on the volume of fibers and testing for correlations with sex and age. (2) Material and Methods: Magnetic resonance imaging of adult patients with diffusion tensor imaging (DTI) was performed on 34 patients. The total number of fibers, volume of UF, and number of tracts were processed using DSI studio software. The DSI studio allows for mapping of different nerve pathways and visualizing of the obtained results using spatial graphics. (3) Results: The total number of UF tracts was significantly higher in the right hemisphere compared to the left hemisphere (right M ± SD = 52 ± 24; left: 39 ± 25, *p* < 0.05). A hook-shaped UF was the most common variant (91.7%). The UF volumes were larger in men (1410 ± 150.7 mm^3^) as compared to women (1325 ± 133.2 mm^3^) (*p* < 0.05). The mean fractional anisotropy (FA) values of the UF were significantly larger on the left side 0.597, while the right UF had an average of 0.346 (*p* < 0.05). Patients older than 50 years old had a significantly higher value of mean diffusivity (MD) (*p* = 0.034). In 73.5% of patients, a greater number of fibers terminated in the inferior part of the inferior frontal gyrus. (4) Conclusions: The morphological characteristics of the UF, unlike the shape, are associated with sex and are characterized by hemispheric dominance. These findings confirm the results of the previous studies. Future research should examine the potential correlation among the UF volume, number of fibers, and total brain volume in both sexes and patient psychological state.

## 1. Introduction

The uncinate fasciculus (UF) was first described by Reil in 1809. It is a white matter tract in the human brain that connects limbic regions in the temporal lobe to the frontal lobe [1,2].

Although the functional role of the uncinate fasciculus is still unknown, the UF is recognized as the major fiber tract connecting the anterior part of the temporal lobe with the orbitofrontal cortex and the frontopolar cortex [3,4,5]. It is attributed to the emotional empathy network and in addition, these nerve fibers are sent to components of the limbic system, including terminating projections to the hippocampus and amygdala [6,7]. The UF as well as the superior longitudinal fasciculus (SLF) and inferior fronto-occipital fasciculus (IFOF) are the main long association fibers in the brain [1,8].

The UF is also known as a ‘hook-shaped’ fascicle connecting the prefrontal cortex and the anteromedial temporal lobe. The traditional anatomical description outlines a temporal stem that hooks around the posterior insula, a subinsular body, and two prefrontal stems that extend to the lateral orbital gyri and the frontopolar cortex [9]. The UF interconnects regions that support acoustic memory, visual information, and emotional responses [10].

UF abnormities are associated with impaired socio-emotional processing and symptom severity in patients with autism spectrum disorder [11].

Tractography is a valuable tool in imaging the central nervous system. Diffusion tensor imaging (DTI) studies have shown that UF is divided into different parts: frontal, insular, and temporal pole [12]. The UF corresponds to the uncus (i.e., Brodmann areas 28, 34, and 36) and the temporal lobe (Brodmann areas 20 and 38) [3,13].

The present study aimed to more accurately describe the anatomical variability of the UF by focusing on the shape of the tract, considering the volume of fibers (with the number of fibers as a side parameter) and testing for correlations with sex and age. We further aimed to present the relationship between the UF in each hemisphere, sex, and age in illustrated and tractography series.

Numerous software packages available allow for an even more robust use of this technique. One example is the DSI studio (http://dsi-studio.labsolver.org) developed by Fang-Cheng. The DSI studio allows for mapping of different nerve pathways and visualizing of the obtained results using spatial graphics.

## 2. Materials and Methods

Patients treated at an academic Neurosurgery Department from 2019 to 2020 were prospectively enrolled into this project.

Participants in this study were 34 adult patients (14 males and 20 females) with an average age of 53.5 years (range: 25–82 years; standard deviation [SD] = 14.69). Handedness was determined by a questionnaire completed by patients (right/left = 29/5). Patients with lesions interfering with the regions of interest (ROIs) or the UF were excluded. Informed consent was obtained from all participants and the study protocol was approved by the local Ethics Committee. The study was conducted in accordance with the Declaration of Helsinki, and the protocol was approved by the Ethics Committee of NKBBN/65/2019.

### 2.1. MRI and DTI Acquisition

Human brains were scanned by MRI (T1, T2-weighed, and DTI with echo planar imaging) using a 20-channel head/neck coil on a single 3.0 T Siemens Magnetom Aera scanner (Erlangen, Germany).

**Figure 1:**
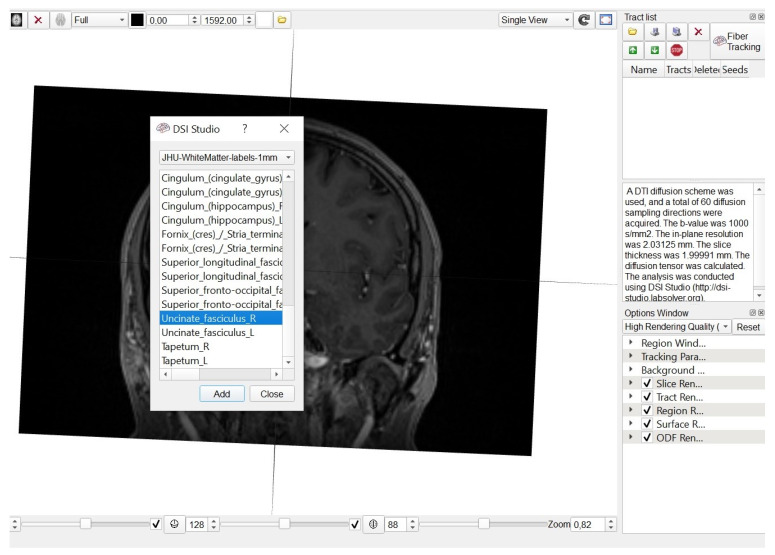
Anatomical atlas loaded in to the DSI studio software.

We used the following DTI acquisition parameters: slice thickness 5.0 mm; matrix—128 × 128; field of view—240 × 240 mm; repetition time—3500 ms; echo time—83.0 ms. Outcome measures of interest included fractional anisotropy (FA), mean diffusivity (MD), and apparent diffusion coefficient (ADC). DTI is most commonly performed using a single-shot, spin-echo, echo planar image acquisition at b-values similar to those used for conventional DWI (typically b = 1000 s/mm^2^). ROIs were defined automatically based on an anatomical atlas loaded into the DSI studio program (Figure 1).

### 2.2. Fiber Tracking

Diffusion tensor images were processed using DSI studio software, BSD License. A DTI diffusion scheme was used and a total of 60 diffusion sampling directions were acquired. The number of directions was based on the experience of previous researchers [14,15,16]. Lebel et al. in their manuscript pointed out that in their study, 30- and 60-direction protocols resulted in either more or longer tracts [16]. The b-value was 1000 s/mm^2^. The in-plane resolution was 1.95 mm. The slice thickness was 2 mm. A deterministic fiber tracking algorithm was used and all of the fiber tracts were determined using the Runge–Kutta algorithm, which was the default for all of the visualizations [17]. The angular threshold was 90 degrees. The step size was 0.98 mm and a total of 200,000 seeds were placed. Fiber trajectories were smoothed by averaging the propagation direction with 30% of the previous directions. Tracts with a length less than 25 mm were discarded. A total of 15,000 tracts were calculated.

### 2.3. Statistical Analyses

Statistica 13 software (Tibco Software Inc., Palo Alto, CA, USA) was used for statistical analysis. The normality of the data distribution was tested qualitatively using the Shapiro–Wilk test. In the event of normal distribution, parametric tests were applied (i.e., Student’s *t*-test for non-dependent and dependent variables and Pearson’s rank correlation test). In the event of non-normal distribution, the data were analyzed using non-parametric tests (i.e., between-group comparisons using the Mann–Whitney test and for dependent variables, the Wilcoxon test; Spearman’s rank correlation test for correlation analysis). All analyses were considered significant at a *p* < 0.05 threshold.

## 3. Results

The UF was identified in both hemispheres in all patients.

**Figure 2:**
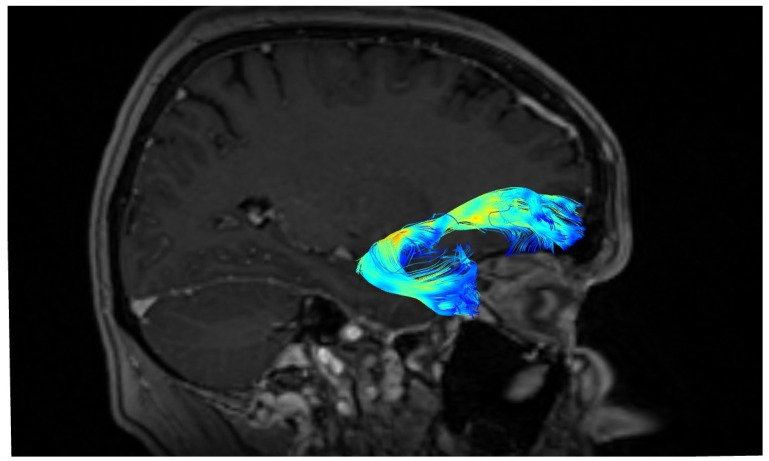
Hook-shape of the uncinate fasciculus (UF).

**Figure 3:**
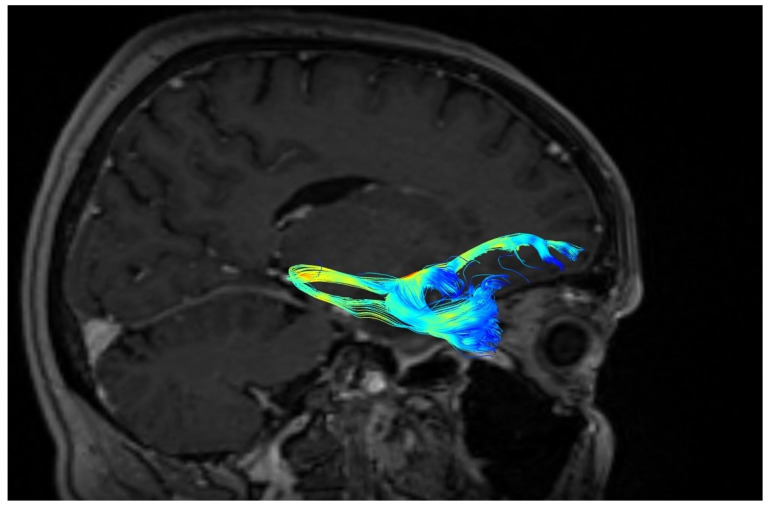
U-shape of the uncinate fasciculus (UF).

**Figure 4:**
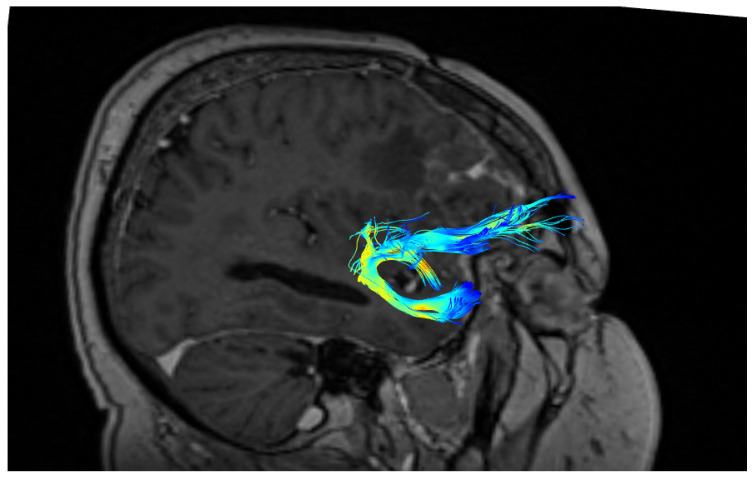
Y-shape of the uncinate fasciculus (UF).

A significantly greater number of UF tracts were observed in the right as compared to the left hemisphere (right: M ± SD = 52 ± 24; left: 39 ± 25, *p* < 0.05). The shapes of the UF in three different patients are presented in Figure 2, Figure 3 and Figure 4. The blue color represents terminating fibers, whereas the green color represents fibers forming the central part of the tract. The colors are selected automatically by the DSI studio program. The hook-shaped (91.7%; *n* = 31, Figure 2), U-shaped (5.8%; *n* = 2, Figure 3), and Y-shaped (2.9%; *n* = 1, Figure 4) were the most common anatomical variants of the UF.

The volume of the UF ranged from 1270 to 1440 ± 170 mm^3^. The volume of the UF was larger in men (1410 ± 150.7 mm^3^) as compared to women (1325 ± 133.2 mm^3^) (*p* < 0.05).

**Figure 5:**
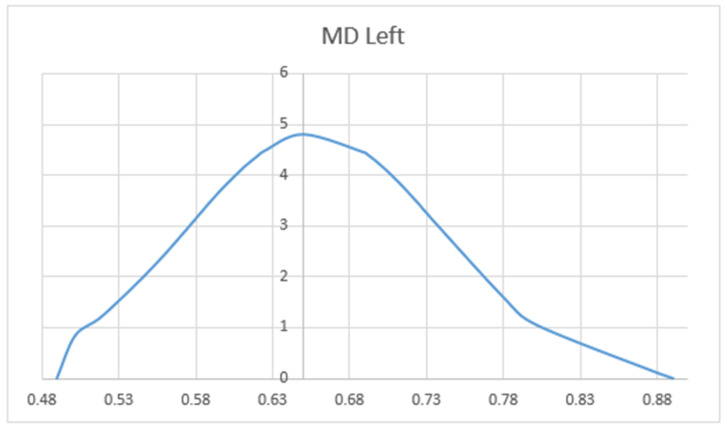
Distribution charts of mean diffusivity (MD) in the left hemisphere of the group of 34 patients aged 25–82.

**Figure 6:**
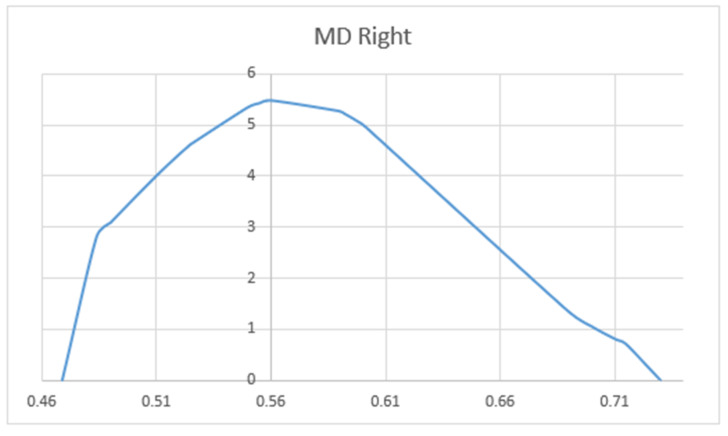
Distribution charts of MD in the right hemisphere of the group of 34 patients aged 25–82.

**Figure 7:**
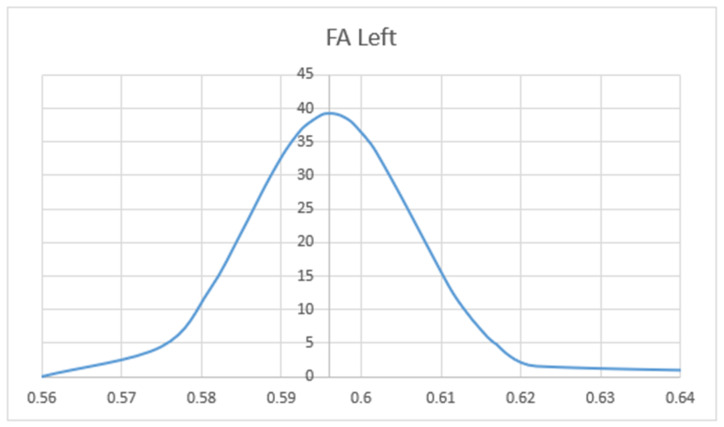
Distribution charts of fractional anisotropy (FA) in the left hemisphere of the group of 34 patients aged 25–82.

**Figure 8:**
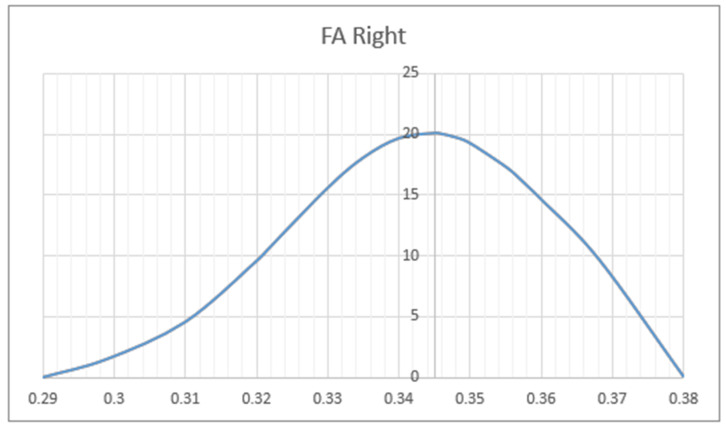
Distribution charts of FA in the right hemisphere of the group of 34 patients aged 25–82.

**Figure 9:**
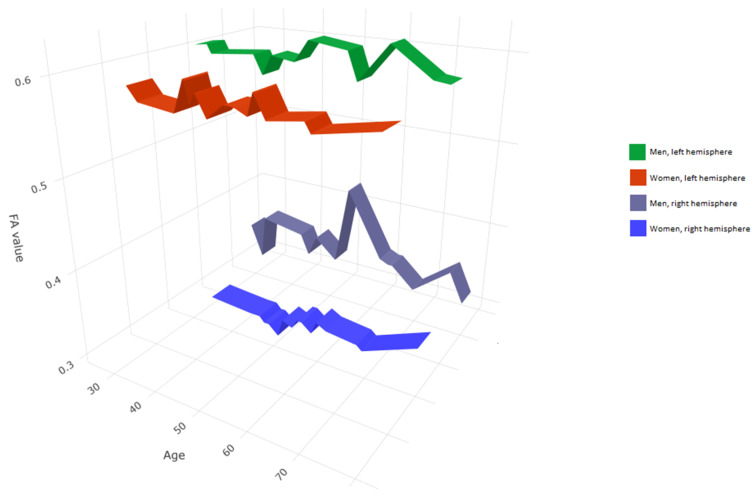
Correlation between FA value, sex, and age in the right and left hemisphere for the group of 34 patients aged 25–82.

We compared the FA and MD values of the UF in terms of hemisphere, sex, and patient’s age. Mean values of FA (MV of FA) were significantly larger on the left side (M = 0.596) as compared to the right side (M = 0.344) (*p* < 0.05). The charts of distributions of FA in the left hemisphere are presented in Figure 5, whereas for the right hemisphere in Figure 6. MD values (distribution presented in the Figure 7 and Figure 8) were significantly higher among patients older than 50 years of age on the left side (*p* = 0.034).In addition, Figure 9 shows a correlation between MV of FA and age for both men and women.

As expected, the analysis of the UF revealed that fibers ended in the frontal and temporal lobes. In 73.5% of patients (*n* = 25 patients), a greater number of fibers terminated in the inferior part of the inferior frontal gyrus (IFG); in 14.7% of patients (*n* = 5), fibers terminated in the gyrus rectus (GyR); and in 11.7% (*n* = 4 patients), fibers terminated in the middle temporal gyrus (MTG).

Factors influencing the variety of UF fibers distribution were analyzed. The total number of UF fibers was not correlated with the ending points of the bundle.

## 4. Discussion

DTI is a modern MRI method that allows for the identification of white matter microstructures [18,19,20]. DTI allows for in vivo spatial visualization of the projection of nerve fibers, and also allows for the determination of the number, lateralization, and direction of the course of the fibers. Several previous reports have demonstrated the multicomponent structure of the UF [4,21,22]. The advent of tractography allowed for one to accurately trace UF fibers. Yagmurlu et al. in their study found that three-dimensional (3D) anatomic organization of the brain tracts is also important in understanding the correct anatomy of the UF, including planning of neurosurgical operations [23].

The role of the UF is still a matter of discussion. Gaffan and Wilson showed that the UF is directly related to the limbic system, which plays an important role in emotion and memory [24].

In light of the current knowledge, the UF is involved in the creation of associative and episodic memory, and also participates in the implementation of socio-emotional and linguistic memory [25]. Changes within the UF are implicated in several mental diseases and disorders, including schizophrenia, anxiety disorders, and Alzheimer’s disease. The results of the DTI analysis may also be used in psychiatry. The use of DTI allows for the visualization of changes in the micro- and macro-structure of white matter in individuals with diagnosed mental disorders [1,3,26,27]. Changes in white matter are reflected in white matter biomarkers such as FA and MD. Several studies have linked lower FA values in the left UF with negative symptoms in schizophrenia, and this pattern was not seen in patients with non-deficit schizophrenia [25,26]. Bahatia et al. demonstrated a reduction in white matter integrity of the UF, which may translate to risk of mental disorders such as first-onset and chronic depression [9]. However, these studies are not equivocal because some researchers have reported an increase or no change in the FA values and indicate a small share of the bundle in the pathogenesis of schizophrenia [28,29]. On the other hand, research involving people with psychopathic personalities consistently demonstrates reduced FA values and significantly increased MD values in the right UF as compared to control subjects [30,31]. In addition, it has been shown that the orbital frontal cortex and temporal pole connection have a smaller volume and are thinner [1,3]. Changes in the UF have also been implicated in the pathogenesis of anxiety disorders. Interestingly, lesions in the UF may result in the occurrence of temporal lobe epilepsy. In addition, the UF is likely associated with behavioral disorders. Indeed, prior studies link anatomical changes in the UF with antisocial behavior and the pathogenesis of Asperger’s syndrome [32,33]. Similar studies of the UF have been conducted in a group of patients with autism spectrum disorder (ASD) [34]. That study demonstrated higher FA values in the left hemisphere in the ASD group as compared to the control group, which may suggest compensatory overgrowth in the ASD [34]. The same study also reported higher FA values in child patients as compared to adult patients [34].

One of the limitations of the study is that the morphology and anatomy of the UF was not correlated with psychological status or personality disorders among patients. Further research should include psychological tests and assessment of the infiltration of the UF by pathological lesions. As in other clinical trials, we showed UF volume asymmetry. Malykhin et al. reported that 71% of patients showed at least 10% asymmetry between the right and left UF, and the average difference between hemispheres was over 40% [35]. Similar conclusions were reached in a post mortem study by Higley et al. [29]. The results of the present study are consistent with these prior studies. The observed hemispheric asymmetry in UF volumes may result from a more consistent or less curly arrangement of the UF fibers in the left hemisphere as compared to the right hemisphere. The value of FA is influenced by many factors, most notably the structural integrity of the fibers, their packing density, degree of myelination, and fiber diameter [25,36,37]. The results of the present study demonstrate that long tracts have significantly higher FA values than anterior tracts. In a post mortem study of normal subjects and patients with schizophrenia, Highley et al. found that the UF is asymmetric in both sexes, such that the UF was 27% larger in the right hemisphere and contained 33% more fibers than in the left hemisphere [29].

Sex differences in the brain are commonly reported. Sexual dimorphism is also reported in studies that use DTI and show differences in the size of the parameters (i.e., volume, FA, and MD), which are most likely caused by differences in the microstructure of white matter between men and women [1,38]. It has been suggested that white matter differences result from different rates of pubertal development, during which there is also dramatic development of white matter in the brain. At the same time, it should be noted that the relative volume of the brain increases faster in boys than girls [39]. It is also worth noting the influence of other, obvious, gender-specific features [38]. For example, men may have a proportionally larger skull and thus, a larger proportion of white matter in the brain as compared to women. On the other hand, the percentage of gray matter in the brain has shown to be higher among women than men [40,41].

During adolescence, FA values and white matter volume gradually increase, which is likely due, in part, to the degree of myelination of nerve fibers [38]. At the same time, after reaching a peak between 28 and 35 years of age, the values of these parameters become inversely proportional to age and therefore, are systematically reduced. This is an expression of brain aging manifested as a loss of myelinated fibers and myelin itself [1,38,39].

Based on the analysis of the collected data, we showed a significant difference in FA values for the left side compared to the right side of the UF (0.596 vs. 0.344). Due to the fact that the group of patients differed both in age and gender, the question arises as to the reason for the difference in FA. It was assumed that a higher FA value reflects a more directed movement of water molecules in nerve fibers, as well as greater coherence of themselves [42]. The asymmetry in the distribution of FA values reflects the difference in the structure and functioning of the UF in both hemispheres of the brain, as well as their different development [42]. The higher value of FA on the left side, compared to the right side, may indicate that it is composed of a greater number of fibers included in the bundle or that their greater density or the arrangement of the fibers is less winding [36,43]. The FA parameter depends on the microstructural properties of nerve fibers; it may indirectly reflect the speed of conduction, thus differences in the values of this parameter may indicate different functioning of the cerebral hemispheres. The obtained results, taking into account the different age and sex structure of the participants, may indicate the lateralization of brain functions [43].

It should be noted that the available literature data are inconsistent with regard to the asymmetric distribution of FA values for the UF. The likely reasons for the discrepancies between the research results are the differences in their methodology, as well as the heterogeneity of the population. Part of the research work showed higher FA values for the UF on the left side [42,43,44,45,46]. However, there are also data in the literature showing rightward FA asymmetry [47] or no asymmetry in FA values at all [48,49]. A 2011 study by Kitamura et al. showed that the FA value for the right (but not left) UF is significantly higher in men as compared to women. This result suggests the presence of sexual dimorphism in the frontotemporal area [41]. A 2015 study by Alm et al. demonstrated higher MD values in both the right and left UF in women as compared to men [50].

In addition, it is important to note that FA and MD values are influenced by the mutual relationship of age and sex, and future data should be collected based on the correlation of these variables. Given the known influence of age and sex, data should be collected on the same MRI scanner and analyzed using a similar tracking algorithm.

Another limitation of the present study was that the UF was measured in patients following a traumatic brain injury (TBI). We found that FA values were higher in the left hemisphere in men as compared to women, and there were no sex differences in MD. Kurki also pointed out that the fibers located in the central part of the UF are characterized by high FA values, which decrease towards the peripherally located fibers [51].

The value of FA is conditioned by many factors. The anatomical aspects of the brain have a significant influence on the parameters of FA tractography. In the work of Hsu et al. the relationship between age, gender, brain anatomy and FA values for 145 adults was analyzed. The results of this analysis proved that the MV of FA is inversely correlated with age. However, the anatomical points where FA was examined were a very important parameter. Hsu et al. while analyzing the anterior part of the corpus callosum, internal capsule and posterior paraventricular region, shown that these anatomical points correlate most with the age-related FA decrease. However, FA values in the temporal and occipital lobes were not correlated with age at all. In the same study, differences between the anatomy of the brain and the gender of patients undergoing FA were also found. Men had higher FA values than women, especially in the right deep temporal regions [52].

In our study, we did not analyze individual anatomical regions in the course of the UF, but subsequent analyses should be carried out based on the exact anatomy of the bundle, taking into account the age and sex of the patients. It should be noted that the accuracy of imaging using DTI depends on the homogeneity of the magnetic field gradients, which translate into the value of the b-matrix. System errors, including equipment-dependent restrictions and scanner or gradient coils, are associated with the spatial heterogeneity of diffusion gradients. [53,54]. The spatial heterogeneity of diffusion gradients, in turn, causes vector distortion and ultimately, falsification of the resulting image. Thus, it became necessary to develop a model that would eliminate the problem as much as possible. The first experimental mathematical scheme to retrospectively correct the effects of spatial gradient field distortions on diffusion-dependent imaging was developed by Bammer et al. [54]. However, to optimally eliminate and correct measurements of the diffusion tensor caused by heterogeneity in the magnetic field gradients, the calibration technique BSD-DTI (b-matrix spatial Distribution DTI technique) was developed and introduced, which significantly improved the fiber tracking procedure. The technique was first introduced in the literature in 2015 by Krzyżak et al. [55]. A year later, Kłodowski et al. conducted a clinical verification that demonstrated the correctness and clinical usefulness of BSD-DTI [56]. Thanks to the introduction of BSD-DTI, it is possible to eliminate conversion errors and account for the spatial distribution of the b-matrix [55,57].

BSD-DTI increases the representativeness and replicability of the obtained results, which allows for comparisons between the obtained images. The BSD-DTI technique allows the correction of not only the magnitude, but also the direction of the diffusion gradient, which is crucial for accurate fiber tracking [53,55].

Fiber tracking has become a valuable method of qualitatively describing many nerve pathways in the brain of a living organism that remain inaccessible to conventional imaging. However, the quantitative assessment of visualized connections has a high risk of false-positive results. Due to the complexity of nerve fibers in the human brain, fiber tracking is undoubtedly subject to a high risk of errors that can arise from the spatial arrangement of the fibers. Crossing, branching, or narrowing of fibers are examples of configurations that can result in identical voxel signals. This is due to the diversity of the spatial arrangement of the white matter fibers, including, for e.g., their intersection. This diversity in fiber arrangement can lead to false lengths of reconstructed fiber tracts [58]. To optimize imaging fidelity, higher-order computational models aimed at precisely eliminating false positive nerve pathways should be used. Despite the introduction of software that creates more and more accurate neuro-anatomical models, in the case of practical use of fiber tracking, the limitations of the method should still be considered [59,60].

In tractography, determining the number of fibers is still a big challenge. Metrics “number of fibers” and “spatial extent of pathways” in direct viewing suggests that the result obtained determines the actual number of white matter fibers identified by axon projections.

These terms are more commonly used in the literature; however, these parameters are not a real numerical representation of the number of fibers and the obtained measurements are associated with a high risk of obtaining a result with an error.

According to data from Jones et al., the reconstruction is influenced by variables such as fiber length, as well as their branching, curvature, and the degree of myelination. Their influence may cause the reconstructed number of fibers to differ from the actual state. Therefore, it should be added that in order to maximize the reliability of quantitative assessments, one should strive to develop procedures correcting the influence of variables on the identification and reconstruction of the actual number of nerve fibers. The authors suggest that when interpreting the obtained results, the term “improvement counter” should be used [58,61]. Tractography has proven to be a useful tool for identifying cortical connections. At the same time, on the basis of measurements obtained by means of probabilistic tractography, it became possible to develop maps of cortical tract length (CTL). This will allow the assessment of which fibers are involved in the projection of distant or local connections. Analysis by Bajada et al. from 2018 provided information on the variability of the distribution of projections of different lengths in the cerebral cortex, as well as the correlation between fiber length and structural features (myelination, cortex thickness) and the relationship between the degree of network complexity at rest and the fiber length profile [62].

## 5. Conclusions

This study presents the anatomy and morphology of the UF. The patient group selected in the study and the criteria used for the research suggest that, under such parameters, the most common UF shape is a hook-shape. Under these same assumptions, the MV of FA was shown to be higher in the left hemisphere as compared to the right hemisphere. The present study demonstrated that morphological characteristics of the UF are associated with sex and are characterized by hemispheric dominance. These findings confirm the results of previous studies. The shape of the UF was not correlated with sex differences nor laterality of the brain hemisphere. Future research should examine the potential correlation between UF anatomy and patient’s psychological state. Future research should also test for correlations among UF volume and total brain volume in both sexes.
